# Differential Expression of Claudin in Odontogenic Cysts

**DOI:** 10.1055/s-0041-1740440

**Published:** 2021-11-22

**Authors:** Ekarat Phattarataratip, Kraisorn Sappayatosok

**Affiliations:** 1 Department of Oral Pathology, Faculty of Dentistry, Chulalongkorn University, Bangkok, Thailand; 2 Department of Oral Diagnostic Sciences, College of Dental Medicine, Rangsit University, Pathum Thani, Thailand

**Keywords:** claudin, odontogenic cyst, odontogenic keratocysts, dentigerous cysts, radicular cysts, calcifying odontogenic cysts

## Abstract

**Objective** This study aimed to analyze claudin-1, -4, and -7 expression in different types of odontogenic cysts (odontogenic keratocysts [OKCs], dentigerous cysts [DCs], calcifying odontogenic cysts [COCs], and radicular cysts [RCs]) as well as its association with OKC recurrence.

**Materials and Methods** Seventy samples of odontogenic cysts samples were immunohistochemically stained to detect claudin-1, -4, and -7 expression. Patient information and OKC recurrence data were recorded. The staining was analyzed semiquantitatively and categorized based on the pattern and percentage of positively stained cystic epithelial cells.

**Statistical Analysis** Expression of different claudins between groups was analyzed using the Kruskal–Wallis test with Dunn's test, followed by post hoc pairwise comparison. The association between claudin expression and OKC recurrence was analyzed by the Mann–Whitney U test. Correlations among claudin expression were examined with Spearman's correlation coefficient. Level of significance was at *p* < 0.005.

**Results** Claudin-1 was widely expressed in every odontogenic cyst. Most DCs (50%) expressed claudin-1 in more than 75% of cells, as did RCs (65%), while most OKCs (50%) expressed claudin-1 in 26 to 50% of cells. Most COCs (50%) expressed claudin-1 in 51 to 75% of cells. Every sample of OKC and RC was positive for claudin-4, but no sample showed staining in more than 51% of cells. Every odontogenic cyst was positive for claudin-7. DCs (35%), OKCs (55%), and RCs (40%) mostly showed staining in 26 to 50% of cells. High claudin-1 expression was shown in COCs, DCs, and RCs, while low expression of claudin-4 was shown in every odontogenic cyst. For claudin-7, the expression is high only in COCs. Claudin-1 and -4 was significantly different among each odontogenic cyst. High expression of claudin-1 was correlated with OKC recurrence. The correlations of claudin-1 with claudin-7 expression and claudin-4 with claudin-7 expression were significant in DCs. In COCs, claudin-1 and claudin-7 expression was significantly correlated.

**Conclusions** The expression of claudin-1, -4, and -7 was present in every odontogenic cyst, but the proportion of positive staining cells was different. Expression of claudin-1 is associated with OKC recurrence. Dysregulation of claudin expression may play a pathogenic role in cyst pathogenesis.

## Introduction

The epithelium is a barrier protecting underlying structures. Alterations of epithelial structures are a first step for many pathologies. Maintenance of epithelial integrity, organization, and permeability is essential for cell proliferation and differentiation. Several proteins are responsible for epithelial polarity. The most important type are tight junction proteins in which claudin proteins are the most important ones. Currently, there are ∼27 claudin members, and most of them are expressed in epithelial cells. 1

Among these, claudin-1, -4, and -7 have been widely studied. Several studies have shown variable expression of claudin-1, -4, and -7 in various lesions, such as odontogenic tumors 2 and oral squamous cell carcinoma (OSCC). 3 4

Claudin-1 is expressed in most tissues. The role of claudin-1 in controlling cellular permeability is clearly shown in claudin-1 knockout mice, which died from severe dehydration. 5 Overexpression of claudin-1 is associated with advanced stage and lymph node metastasis in OSCC. 3

Claudin-4 also regulates cellular permeability. Low expression of claudin 4 is related to lymphangiogenesis in gastric cancer. 6 Low claudin-4 expression is associated with poor prognosis in breast carcinoma *in situ* and breast cancer. 7 However, its expression does not correlate with staging in OSCC. 3

Claudin-7 is also expressed in a variety of epithelial tissues. Claudin-7 knockout mice show extensive inflammation, pathologic hyperplasia, and adenoma in intestinal tissue. 8 Claudin-7 has been suggested to be a tumor suppressor gene involved in the development of colorectal cancer. 9 Loss of claudin-7 expression is associated with high pathologic grade and advanced staging in OSCC. 4

Odontogenic cysts are an important group of lesions in jaw bone that are responsible for ∼7 to 15% of all oral and maxillofacial biopsies. 10 11 Most odontogenic cysts do not recur, except odontogenic keratocysts (OKCs), for which the recurrence rate can be up to 58.3%. 12 Main component of odontogenic cysts is cystic epithelium and most odontogenic cysts are derived from epithelial rest. Odontogenic cyst growth is believed to be from permeability changes in the cystic epithelium after epithelial rest is stimulated. There was only one study on odontogenic cyst and claudin that showed limited expression of claudin in dentigerous cysts (DCs), OKCs, and radicular cysts (RCs), but the correlation of claudin expression and OKC recurrence was not revealed. 13 And there was no data on claudin expression in calcifying odontogenic cysts (COCs). Study from Bello et al 14 showed that claudin-1 and -7 were strongly expressed in enamel epithelium and ameloblast, while expression of claudin-4 was very weak. Previous study from our group demonstrated that claudin-1 is highly expressed in odontogenic epithelium of ameloblastic fibroma but was weak in ameloblast-like cell and stellate reticulum-like cells in ameloblastoma. 2

The objectives of this study were to analyze claudin-1, -4, and -7 expression in different types of odontogenic cysts, COCs, DCs, OKCs, and RCs, as well as its association with OKC recurrence.

## Materials and Methods

Seventy samples of odontogenic cysts samples (10 COC, 20 DC, 20 OKC, and 20 RC) from archived formalin-fixed paraffin-embedded blocks housed in the Department of Oral Pathology, Chulalongkorn University) were used in the study. The diagnosis was re-examined by two board-certified oral pathologists based on World Health Organization criteria of odontogenic cyst histopathology. Patient information, including age, sex, anatomical site, and OKC recurrence (7 years follow-up), was recorded. The study was approved by the Human Research Ethics Committee, Faculty of Dentistry, Chulalongkorn University.

### Immunohistochemistry

Bond-Max Autostainer (Leica Microsystems) was used for immunohistochemistry. Five-micrometer-thick sections were deparaffinized with Bond Dewax Solution. Antigen retrieval was performed by incubating the slides at 95°C for 30 minutes with Bond Epitope Retrieval Solution (for claudin-1 and -7). The incubation time was changed to 20 minutes at 95°C for claudin-4. The primary antibodies used were polyclonal anticlaudin-1 (1:200 dilution), monoclonal anticlaudin-4 (1:500 dilution), and monoclonal anticlaudin-7 (1:500 dilution) antibodies (Invitrogen, Camarillo, California, United States). A Bond Polymer Refine Detection kit (Leica Microsystems, Germany) was used as a polymer detection system. Hydrogen peroxide (3%) was applied for 5 minutes to block endogenous peroxidase activity. The primary antibodies for each claudin were applied. Then the slides were incubated for 50 minutes at room temperature, followed by 12-minute incubations with post primary polymer and polymer poly-horseradish peroxidase immunoglobulin G. The sections were reacted with diaminobenzidine solution for 3 minutes and counterstained with hematoxylin. In each step, Bond Wash Solution was used as a rinsing buffer. As positive controls, colonic mucosa samples were used. Negative controls were prepared using isotype-matched antibodies.

### Interpretation of Immunohistochemistry and Statistical Analysis

The sections were evaluated under a Nikon Eclipse 800 microscope (Nikon Corporation, Japan). Only the plasma membrane of the cystic epithelial lining was regarded as having positive claudin staining. Positive staining for claudin was semiquantitatively evaluated independently by two of the authors who were blinded to the clinicopathological data. The pattern of positive cystic lining cells for each claudin was also analyzed. The score was reported as the percentage of positive cystic epithelial cells. The samples were classified as follows: immunostaining was considered negative or scored 0 when none of the cystic epithelial cells were positively stained; immunostaining was scored 1+ when ≤ 25% of cystic epithelial cells showed positive staining; immunostaining was scored 2+ when between 26 and 50% of cystic epithelial cells showed positive staining; immunostaining was scored 3+ when between 51 and 75% of cystic epithelial cells showed positive staining; and immunostaining was scored 4+ when more than 75% of cystic epithelial cells showed positive staining. The slides were randomly reviewed to minimize possible bias.

For specific comparisons, the expression levels were further grouped into low expression (scores 0 and 1 + ) and high expression (scores 2 + , 3+ and 4 + ).

The results were statistically analyzed using IBM SPSS Statistics version 22 (IBM Corporation, New York, United States) for Windows. Continuous variables are expressed as the means ± standard deviation. Comparative analyses of the expression of different claudin proteins between groups were performed using the Kruskal–Wallis test with Dunn's test, followed by post hoc pairwise comparison using the Bonferroni method. The association between claudin expression and OKC recurrence in patients was analyzed by the Mann–Whitney U test. Correlations among claudin expression levels were examined with Spearman's correlation coefficient. A *p* -value less than 0.05 was considered statistically significant.

## Results

Demographic data of patients enrolled in the study are shown in Table 1 . Most COCs, DCs, and OKCs occurred in the posterior mandible, while RCs mostly occurred in the anterior maxilla. The average ages of patients with COCs, DCs, OKCs, and RCs were 20.90 ± 7.78, 24.75 ± 14.14, 37.75 ± 26.16, and 37.90 ± 13.43 years, respectively, and the male to female ratios were 2.3:1, 3:1, 1.86:1 and 2.33:1.

**Table 1:** Patient characteristics

Cysts	Gender		Age		Location			
					Maxilla		Mandible	
	Male	Female	Mean ± SD	Range	Ant	Post	Ant	Post
COC	7	3	20.9 ± 7.78	13–40	3	0	1	6
DC	15	5	24.75 ± 14.14	11–34	4	0	0	13
OKC	13	7	37.75 ± 26.16	17–66	0	6	1	13
RC	14	6	37.9 ± 13.43	12–61	10	4	1	5

Abbreviations: COC, calcifying odontogenic cyst; DC, dentigerous cyst; OKC, odontogenic keratocyst; RC, radicular cyst; SD, standard deviation.

**Fig. 1:**
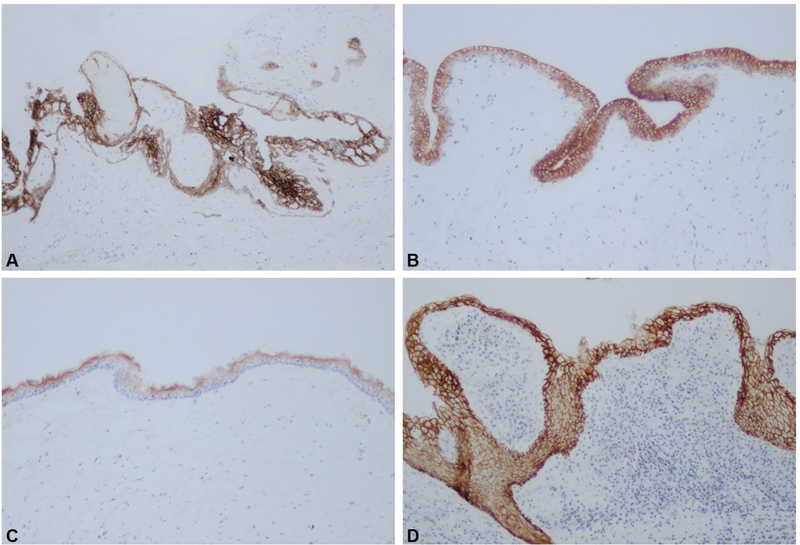
Immunohistochemical reactivity of claudin-1 in calcifying odontogenic cyst ( **A** ), dentigerous cyst ( **B** ), odontogenic keratocyst ( **C** ), and radicular cyst ( **D** ) at X10 magnification.

**Fig. 2:**
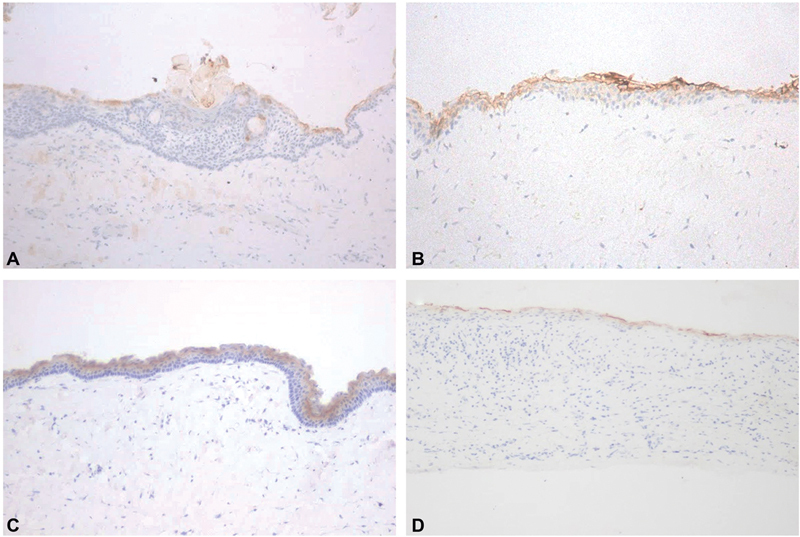
Immunohistochemical reactivity of claudin-4 in calcifying odontogenic cyst ( **A** ), dentigerous cyst ( **B** ), odontogenic keratocyst ( **C** ), and radicular cyst ( **D** ) at X10 magnification.

**Fig. 3:**
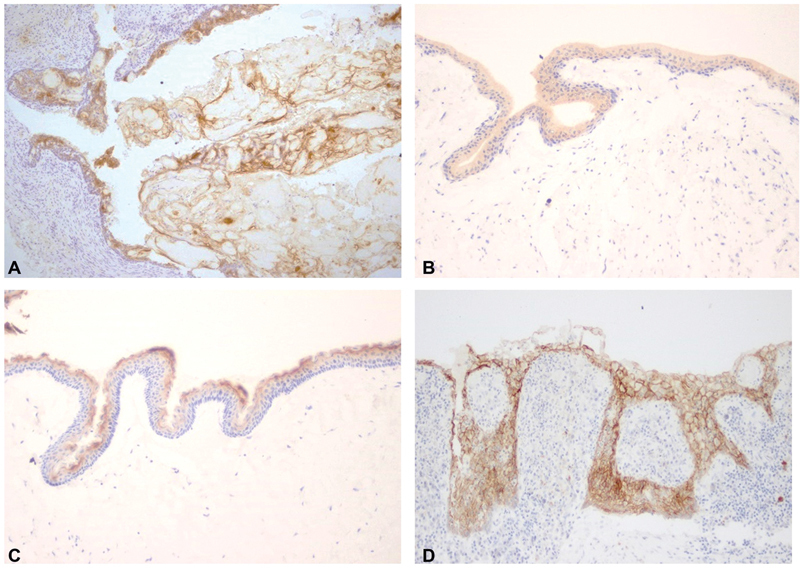
Immunohistochemical reactivity of claudin-7 in calcifying odontogenic cyst ( **A** ), dentigerous cyst ( **B** ), odontogenic keratocyst ( **C** ), and radicular cyst ( **D** ) at X10 magnification

Immunohistochemical reactivities of claudin-1, -4 and 7 in COC, DC, OKC and RC are shown in Figs. 1 , 2 , and 3 , respectively. The levels of immunohistochemical staining for each claudin for each odontogenic cyst type are shown in Table 2 . Claudin-1 and -4 expression among odontogenic cyst was statistically significantly different. Claudin-1 expression between COCs and RCs, COCs and OKCs was significantly different by Bonferroni analysis. Claudin-4 expression between COCs and DCs, COCs and OKCs, COCs and RCs was also significantly different. High claudin-1 expression was shown in COCs, DCs, and RCs, while low expression of claudin-4 was shown in every odontogenic cyst. For claudin-7, the expression was high only in COCs. The relationships between the expression of each claudin and OCK recurrence in patients are shown in Table 3 .

**Table 2:** Levels of claudin-1, -4, and -7 expression in odontogenic cysts

Claudins	Odontogenic cysts	Immunohistochemical staining, *n* (%)					Expression levels, *n* (%)		*p* -Value
		Level 0	Level 1+	Level 2+	Level 3+	Level 4+	Low	High	
Claudin-1	COC* ^,^ °	0 (0)	1 (10)	3 (30)	5 (50)	1 (10)	4 (40)	6 (60)	0.002
	DC	0 (0)	0 (0)	7 (35)	3 (15)	10 (50)	7 (35)	13 (65)	
	OKC*	0 (0)	2 (10)	10 (50)	5 (25)	3 (15)	12 (60)	8 (40)	
	RC°	0 (0)	0 (0)	3 (15)	4 (20)	13 (65)	3 (15)	17 (85)	
Claudin-4	COC ^α,β,€^	5 (50)	5 (50)	0 (0)	0 (0)	0 (0)	10 (100)	0 (0)	0.000
	DC ^α^	1 (5)	7 (35)	8 (40)	4 (20)	0 (0)	15 (75)	4 (25)	
	OKC ^β^	0 (0)	4 (20)	16 (80)	0 (0)	0 (0)	20 (100)	0 (0)	
	RC ^€^	0 (0)	12 (60)	8 (40)	0 (0)	0 (0)	20 (100)	0 (0)	
Claudin-7	COC	0 (0)	1 (10)	2 (20)	7 (70)	0 (0)	3 (30)	7 (70)	0.759
	DC	0 (0)	5 (25)	7 (35)	3 (15)	5 (25)	12 (60)	8 (40)	
	OKC	0 (0)	3 (15)	11 (55)	2 (10)	4 (20)	14 (70)	6 (30)	
	RC	0 (0)	3 (15)	8 (40)	3 (15)	6 (30)	11 (55)	9 (45)	

Abbreviations: COC, calcifying odontogenic cyst; DC, dentigerous cyst; OKC, odontogenic keratocyst; RC, radicular cyst.

Note: Analyses of comparison were performed using Kruskal–Wallis test.

Cyst labeled by letters * ^,^ ° ^,α β,€^ showed statistically significant differences (paired) in the expression of the designated claudins using post hoc pairwise comparison, Bonferroni method.

**Table 3:** Relationship between the claudin expression and the recurrence status of odontogenic keratocyst patients

Odontogenic keratocyst		Immunohistochemical staining, *n* (%)					Expression levels, *n* (%)		*p* -Value
		Level 0	Level 1+	Level 2+	Level 3+	Level 4+	Low	High	
Claudin-1	Recurrence (10)	0 (0)	0 (0)	2 (20)	5 (50)	3 (30)	2 (20)	8 (80)	0.001 a
	No recurrence (10)	0 (0)	2 (20)	8 (80)	0 (0)	0 (0)	10 (100)	0 (0)	
Claudin-4	Recurrence (10)	0 (0)	1 (10)	9 (90)	0 (0)	0 (0)	10 (100)	0 (0)	–
	No recurrence (10)	0 (0)	3 (30)	7 (70)	0 (0)	0 (0)	10 (100)	0 (0)	
Claudin-7	Recurrence (10)	0 (0)	1 (10)	5 (50)	1 (10)	3 (30)	6 (60)	4 (40)	0.628
	No recurrence (10)	0 (0)	2 (20)	6 (60)	1 (10)	1 (10)	8 (80)	2 (20)	

Abbreviation: OKC, odontogenic keratocyst.

a Statistically significant between OKC recurrence and claudin expression.

### Claudin-1

Claudin-1 was widely expressed in every odontogenic cyst. Most DCs (50%) expressed claudin-1 in more than 75% of cells (65%), while most OKCs (50%) expressed claudin-1 in 26 to 50% of cells. Most COCs (50%) expressed claudin-1 in 51 to 75% of cells. The RCs that showed the 4+ staining pattern for claudin-1 showed uniform staining in in every cell layer, while the RCs that showed the 2+ and 3+ patterns showed staining of cystic epithelial cells located within basal cells and the intermediate cystic epithelial layer. These differences in staining patterns were also seen in DCs. In COCs, most of the intermediate cell layer in the cystic lining stained positive for claudin-1. Strong claudin-1 immunoreactivity was noted in ghost cells. In OKCs, most cells in the keratin layers and intermediate cells were positive for claudin-1. High expression of claudin-1 was correlated with OKC recurrence ( *p* = 0.001) ( Table 3 ).

### Claudin-4

Every sample of OKC and RC was positive for claudin-4, but no sample showed staining in more than 51% of cells. In COCs, the cells that stained positively were mainly ghost cells and half of the cases were negative for claudin-4 in which no sample showed staining in more than 26% of cells. Most DCs (40%) and OKCs (80%) showed positive staining in 26 to 50% of cells. In OKCs, the keratin layer stained positively in every sample. Only one DC sample showed negative staining for claudin-4. In RC, every sample was positive, but no sample showed positive staining in more than 50% of cells; most samples (60%) showed positive staining in less than 25% of cells, and the uppermost cystic epithelial cells and intermediate layer cells were the ones that showed positive staining in these samples.

### Claudin-7

Every odontogenic cyst was positive for claudin-7. DCs (35%), OKCs (55%), and RCs (40%) mostly showed staining in 26 to 50% of cells. Most COCs (70%) showed positive staining in 51 to 75% of cells. Strong claudin-7 immunoreactivity was noted in ghost cells, as was seen for other claudins. In OKCs, cells in the keratin layer and intermediate cells were consistently positive for claudin-7, as was seen for claudin-1. High expression of claudin-7 was not correlated with OKC recurrence ( Table 3 ).

### Correlations between the Expression of Different Claudins in Odontogenic Cysts

Table 4 shows the Spearman correlation coefficients between the expression of different claudins in the odontogenic cyst types. The correlations of claudin-1 with claudin-7 expression ( *p* = 0.005) and claudin-4 with claudin-7 expression ( *p* = 0.004) were significant in DCs. In COCs, claudin-1 and claudin-7 expression was significantly correlated ( *p* = 0.005).

**Table 4:** Correlation among claudin-1, -4, and -7 expression in odontogenic cysts

Cysts	Claudin expression	Spearman's rho	*p* -Value
COC	Claudin-1, Claudin-7	0.082	0.005 a
DC	Claudin-1, Cluadin-4	0.367	0.112
	Claudin-1, Claudin-7	0.599	0.005 a
	Claudin-4, Claudin-7	0.612	0.004 a
OKC	Claudin-1, Cluadin-4	0.105	0.658
	Claudin-1, Claudin-7	−0.065	0.786
	Claudin-4, Claudin-7	0.251	0.286
RC	Claudin-1, Claudin-7	0.380	0.098

Abbreviations: COC, calcifying odontogenic cyst; DC, dentigerous cyst; OKC, odontogenic keratocyst; RC, radicular cyst.

a Statistically significant correlation between claudin expression in each cyst.

## Discussion

Our report is the first to study the expression of claudin-1, -4, and -7 in every notable type of odontogenic cyst. Every type of odontogenic cyst had low expression of claudin-4, but claudin-1 and -7 were expressed in every odontogenic cyst. Changes in the cystic epithelium allow fluid movement to generate hydrostatic forces to induce cyst growth, especially for DCs and RCs. Barrier permeability changes in the epithelium are associated with claudin expression. Study by Alvarez et al has reported the involvement of claudin-1 in both transepithelial and paracellular transport, 15 highlighting its importance in tight junction barrier functions. Other claudin is also associated with permeability but with different roles depending on the condition. 1 8 Claudin-1 was highly expressed in COCs, DCs, and RCs, which is different from the previous study. 13 However, the expression was not as strong as that in the positive control. It may be implied that some loss of expression of claudin-1 in cystic epithelium may occur during permeability changes in cystic pathogenesis. This data is correlated with study in ameloblastoma showing that loss of claudin-1 expression is significant in ameloblastoma. 2

OKCs growth is associated with the neoplastic potential that is different from other odontogenic cysts. 12 A study showed that matrix metalloproteinase (MMP) and RANK expression reflects osteoclastogenesis and neoplastic behavior of OKCs. 16 Our study showed that high expression of claudin-1 was correlated with OKC recurrence. Therefore, the expression of claudin-1 in OKCs may reflect the aggressive nature of this type of cyst, similar to that seen in OSCC. 3 A study showed that the expression of claudin-1 is associated with high pathologic grade, perineural and vascular invasion, regional lymph node involvement, and advanced tumor, node, metastasis (TNM) stage. 3 It has been shown previously that the invasive activity of OSCC cells is enhanced by claudin-1 through activation of MMP-1 and -2, resulting in increased cleavage of laminin-5 chains. 17 However, previous study on odontogenic tumors showed weak expression of claudin-1 in ameloblastomas and low expression of claudin-1 in ameloblastoma were significantly associated with tumor recurrence 2 that is different from OKC in our study.

The upregulation or downregulation of claudin-1 and the impact of clinical or pathologic parameters seem to depend on the type of pathology. For example, the claudin-1 expression level in breast cancer differs depending on the subtype of cancer. Expression of claudin-1 is significantly higher in the poor prognosis breast cancer than in other subtypes. Progression of breast cancer is associated with claudin-1 expression affecting Ephrin B1 and EpCAM. 18 Claudin-1 also shows exhibition of antiapoptotic effects in some breast cancer cell lines, like MCF-7. 19 However, some studies have shown a correlation of increased malignancy, invasiveness, and recurrence of breast cancer with total or partial loss of claudin-1 expression. 20

Study in hepatocellular carcinoma demonstrated that increased expression of claudin-1 is involved in epithelial to mesenchymal transition during early carcinogenesis. 21 Nevertheless, in another study, reduced expression of claudin-1 was reported to be a marker for poor prognosis in hepatocellular carcinoma. 22

In pancreatic cancer, increased expression of claudin-1 was found to be associated with tumor aggressiveness. The mechanism tying claudin expression to the aggressiveness of pancreatic tumors may be from the claudin-1-induced activation of mitogen-activated protein kinase 2 and cell dissociation in pancreatic cancer cells. 23

In melanoma, claudin-1 is abnormally expressed in the cytoplasm of malignant cells and not in the cell membrane. This phenomenon may be related to the influence of claudin-1 on protein kinase-C (PKC) activity. PKC activation causes an increase in the transcription and protein expression of claudin-1 and thus cell motility. 24 Melanoma cells transfected with claudin-1 show increased secretion of MMP-2, reflecting the contribution of claudin-1 to the cell invasion process. 24

The expression of claudin-4 in every cyst type was lower than that of claudin-1. In OKCs, most positive staining was seen in the keratin layer; this was similar to the pattern seen in COCs, which showed most positive staining in the uppermost ghost cell layer. In RCs, the uppermost cystic epithelial cells and intermediate layer cells were positive. The loss of claudin-4 seen in RCs and DCs may reflect their role in cyst growth. Claudin-4 is usually upregulated in cancers and shows a correlation with clinical parameters, 25 26 but in our study, it was not associated with OKC recurrence. The effects on permeability of claudin-4 *in vitro* depend on the cell type investigated; claudin-4 acts either as a general barrier or as a Na ^+^ barrier without affecting Cl ^-^ permeability. 27 Claudin-4 is downregulated under various conditions that cause increased permeability. 28 Claudin-4 is minimally expressed during human late bell stage tooth development in the outer enamel epithelium and stellate reticulum. Therefore, it may not have the same role in the pathogenesis of odontogenic cysts as it does in odontogenic tumors. 14

Every odontogenic cyst type was positive for claudin-7, but the proportion of positive cells was less than that seen for claudin-1. In OKCs and COCs, strong immunoreactivity was shown in the keratin layer and ghost cells, as was seen for claudin-4. The study of claudin-7 showed that loss of expression of claudin-7 is associated with pathologic grade, advanced TNM stage, large tumor size, the presence of microscopic perineural and vascular invasion and regional lymph node involvement. 3 However, in OKCs, there was no correlation between the expression of claudin-7 and recurrence of OKCs.

Studies on the effects of claudin-7 on permeability have also produced controversial results. Alexandre et al 29 found that overexpression of claudin-7 in LLC-PK cells caused a decrease in Cl ^-^ permeability and a simultaneous slight increase in Na ^+^ permeability. On the other hand, Hou et al 30 found that knockdown of claudin-7 in MDCK and LLC-PK cells increased Na ^+^ permeability and decreased Cl ^-^ permeability.

*In vitro* studies suggested that dysregulation of claudin expression may play a pathogenic role in many diseases, 2 3 4 8 9 14 20 25 26 29 but the mechanisms seemed to be poorly understood and varied between different cancers and claudin isoforms. Also, the roles of tight junction proteins in diseases pathogenesis are complex process. Many signaling cascades are involved including cell differentiation, 31 cell proliferation, 32 or even angiogenesis process. 33 These aspects cannot be answered by immunohistochemical study. Further study should be conducted to find the answer of odontogenic cyst pathogenesis involving tight junction protein function and their roles in cystic epithelium permeability in cyst growth as well as their effects on cyst behavior.

## Conclusion

Claudin protein may have a role in odontogenic cyst pathogenesis. The expression of claudin-1, -4, and -7 was present in every odontogenic cyst type, but the proportion of cells with positive staining was different. Claudin-1 expression is associated with recurrence behavior of OKC.
